# In vitro marginal and internal adaptation of four different base materials used to elevate proximal dentin gingival margins

**DOI:** 10.4317/jced.59652

**Published:** 2022-07-01

**Authors:** Hoda S. Ismail, Ashraf I. Ali, Rabab El. Mehesen, Franklin Garcia-Godoy, Salah H. Mahmoud

**Affiliations:** 1 Operative Dentistry Department, Faculty of Dentistry, Mansoura University, Mansoura, Egypt; 2 Department of Bioscience Research, College of Dentistry, University of Tennessee Health Science Center, Memphis, Tennessee, USA; 3 The Forsyth Institute, Cambridge, MA, USA

## Abstract

**Background:**

There is still debate about the most appropriate restorative material category to relocate the proximal deep cervical margins, thus, this study aimed to compare the marginal and internal adaptation of four base materials used for deep margin elevation, and to evaluate each base material/overlying composite interface.

**Material and Methods:**

Fifty six molars received class II cavities with dentin/cementum gingival margins. They were divided into four groups and their gingival margins were elevated using either; resin modified glass ionomer (RMGI), highly viscous conventional glass ionomer (HV-GIC), flowable bulk fill resin composite (Bulk Flow) and bioactive ionic resin (Activa). The rest of the cavities were completed with the same overlying composite. Half of each group was either; kept in sterile water for 1 week, or subjected to 18 months water storage and 15,000 thermal cycles. Base materials/gingival dentin interfaces were examined under a scanning electron microscope at different magnifications, and percentage of continuous margin (% CM) and maximum gap width (MGW) were analyzed, in addition to base materials/overlying composite interfaces evaluations. % CM values were statistically analyzed using Two-way analysis of variance, Tukey post hoc tests (at *p*<0.05) and Pearson’s correlation while MGW values were analyzed using Kruskal–Wallis, Mann–Whitney U tests and Spearmen correlation

**Results:**

Both Bulk Flow and Activa had better marginal integrity than RMGI and HV-GIC. All base materials were adversely affected by aging. All base materials/overlying composite interfaces were continuous and age defying.

**Conclusions:**

In terms of marginal integrity, Bulk Flow and Activa might be preferable for proximal dentin margin elevation under direct restoration compared to the other tested base materials.

** Key words:**Deep proximal margin, interface analysis, marginal quality, open sandwich technique.

## Introduction

The current adhesive technology and modern resin composite materials allow for the restoration of severely damaged teeth using directly placed resin composite restorations (1). Subgingival cavities with cervical margins extending below the cemento-enamel junctions (CEJ) generate significant technical and operative challenges including loss of partial or total sealing of the cervical margins in the absence of enamel (2). Adhesion to dentin is challenging due to the high organic component, tubular structure, and permeability, along with the low surface energy of dentin (3). Consequently, bonding to deep cervical dentin and keeping the margins of any adhesive restoration sealed throughout the time could not be considered entirely predictable and safe (4).

Elevation of proximal dentin margins under either direct or indirect restorations have been investigated using either glass ionomer (GI)-based or resin-based materials (5,6). Several studies advocated the use of resin composites for bonding with such margins (7,8), especially with the current types of adhesives (7). Among different promising current resin composites while dealing with such margins, is bulk fill resin composites that could be placed in layers up to 4-mm in thickness and cured in 1 single step (9). Thus, they can be quickly applied and save chair time, especially when used for deep and large cavities (9). Regardless of the previous data, some recent literature argue that GI with its hydrophilic nature, flexibility, chemical bonding and enhanced mechanical and wear properties of their newer generations, could be a more suitable option for bonding to deep moist dentin/cementum margins (10,11). Another possible promising restorative option for elevation of such margins is bioactive restorative materials. They are a relatively new category of materials which react to pH changes in the mouth by uptaking calcium, phosphate, and fluoride ions to maintain the chemical integrity of the tooth structure (12). In addition, they are moisture friendly and delivered with relatively low viscosity that may be useful while bonding and adapting to deep gingival margins (12).

Assessing the external and internal marginal integrity of restorative materials could be considered as important parameters when predicting its long term behavior (13). In case of laminate restorations, the intimate and durable adaptation between each base material and overlying resin composite ensures good stress transmission and long term success (14). Regardless of the previous data, there is still debate in the current literature about the most appropriate restorative material category to relocate the proximal deep cervical margins, especially after long term aging. Furthermore, there is no enough information about bonding ability of resin composites to new generations of GI or bioactive restorative materials. Therefore, the aim of this study was to evaluate and compare the marginal and internal adaptation of 4 different base materials used to elevate the proximal dentin gingival margins, in addition, to qualitatively evaluate the interface between each base material and overlying resin composite. The research hypotheses were: (1) the type of base material and the aging condition would not affect the external nor internal marginal adaptation when each base material was bonded to dentin gingival margin; (2) both external and internal adaptation values of each base material would correspond to each other; (3) there would be no difference in the qualitative evaluation of the interface between each base material with the overlying resin composite.

## Material and Methods

-Materials 

Four commercially available restorative materials were tested in the current study. Resin modified glass ionomer (Fuji II LC) (RMGI), highly viscous conventional glass ionomer (EQUIA Forte Fil) (HV-GIC), flowable bulk fill resin composite (Tetric N-Flow Bulk Fill) (Bulk Flow) and bioactive ionic resin (Activa Bioactive Restorative) (Activa). The detailed description of the materials is presented in Table 1.

**Table 1:**
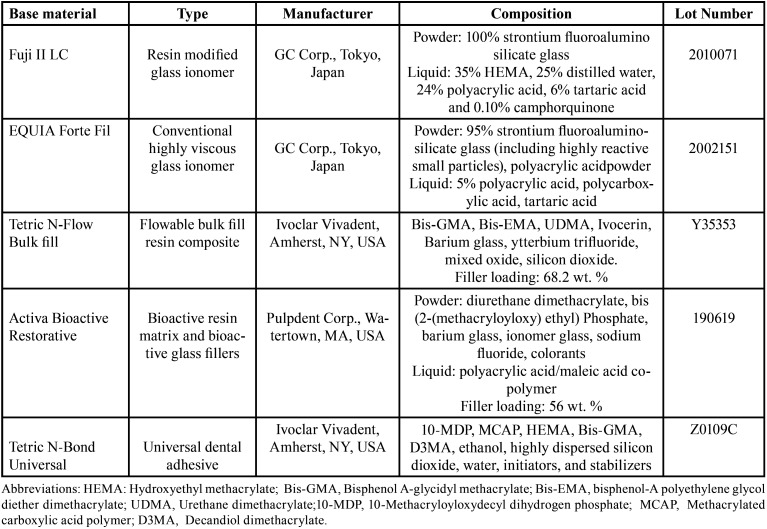
Materials used in the study.

-Cavity preparation 

Fifty six human upper molars recently extracted due to periodontal disease were included in this study; they had approximately similar dimensions, with no caries or cracks and stored in 0.1% thymol solution until used. The teeth were collected after approval of the Ethics Committee No. (A101111219).

Roots of all teeth were fixed vertically in acrylic resin cylinders up to 2 mm below CEJ to facilitate the preparation and restoration steps. Compound class II cavities with standardized dimensions were prepared on the mesial surfaces of all teeth using cylindrical medium grit diamond bur (K881 012, öko DENT, Germany) and finished with a 25 µm finishing diamond under copious water coolant with a high speed handpiece (W&H, RC-90RM, Austria). The cavity dimensions were: occlusal: 3.0 mm bucco-lingual width, 3.0 mm depth; box: 1.0 mm below CEJ, 1.5 mm mesio-distal dimension at the bottom, 3.0 mm bucco-lingual width (15). The margins were not beveled with slightly rounded line angels. New burs were used after preparation of every five cavities. Buccal and palatal walls of the proximal boxes of all teeth were marked with pencil 1.0 mm above CEJ (to mark the level of the base material).

Teeth were randomly assigned into four different groups, 14 molars each, according to the base material used. Each group’s teeth were numbered from 1 to 14 with specific color for each material group.

-Restorative procedures

After preparation procedures, cavities were washed with water and dried. For RMGI and HV-GIC groups, the gingival, buccal, palatal and axial dentin margins of the cavities were conditioned as recommended by the manufacturer with dentin conditioner (GC Co., Tokyo, Japan) for 20 seconds, followed by rinsing and drying. Occlusal and proximal enamel margins of all groups’ cavities were selectively etched with 37% phosphoric acid (N-Etch, Ivoclar Vivadent, Amherst, NY, USA) for 15 s, rinsed with water for the same time, gently dried with oil free air without desiccation. For Bulk Flow and Activa groups, a universal adhesive (Tetric N-Bond Universal, Ivoclar Vivadent) was applied before base material placement on all cavity surfaces, air thinned and light cured as recommended by the manufacturers’ instructions with a LED curing light (Elipar Deep Cure, 3M ESPE, St. Paul, MN, USA) operating at 1,000 mW/cm2, checked periodically after every 5 samples with a radiometer (Bluephase Meter II, Ivoclar Vivadent).

The restorations were performed as two steps, one for the base material application and the second for restoring the remaining cavity. A Tofflemire matrix-band (Fintrec dead soft matrix, Pulpdent Corp., MA, USA) was contoured and placed around each tooth, making sure that the end of the band was beyond the gingival margin of the cavity to prevent the creation of gross marginal discrepancies. After that, each group was restored up to 1.0 mm above CEJ using the group specific base material in a bulk technique. All base materials were mixed, dispensed and cured (RMGI, Bulk Flow and Activa groups) according to the manufacturers’ instructions. The Tofflemire matrix band was changed to another circumferential matrix system (No. 2162, HAWE SuperMat, Kerr Corp., Orange, CA, USA). For RMGI, HV-GIC and Activa groups, the universal adhesive was applied after base material placement with the same technique mentioned earlier. The remaining cavity was restored with a nanohybrid regular bulk fill resin composite material (Tetric N-Ceram Bulk Fill, Ivoclar Vivadent) that was inserted in the cavity in 2 horizontal increments using a plastic instrument (Artman Instruments, GA, USA). Each increment was cured from the occlusal surface for 20 seconds. Additional curing for 20 seconds was performed from the proximal surface after removal of the matrix-band.

All specimens were stored in distilled water at 37°C for 24 hours in an incubator prior to the finishing and polishing procedures. Finishing and polishing of the restorations and removal of any visible overhangs were performed with Al2O3 discs (Extra-Thin Sof-Lex discs, 3M ESPE) using a low speed handpiece (Strong 204, Daegu, South Korea) under water cooling. All specimens were removed from their fixation blocks and received ultrasonic cleaning before further testing steps. All preparation and restoration procedures were performed by a single operator using magnification (×4 loupes, Amtech, Wenzhou, China) and LED head light illumination.

-Artificial aging

Each material group (n = 14) was randomly divided into 2 subgroups, according to aging conditions either; (1) kept in distilled water for 1 week at 37°C, or (2) stored in sterile water for 18 months at 37ºC in an incubator and changed weekly (16), then thermo cycled for a total number of 15,000 cycles (SD mechatronik thermocycler, Germany) which approximately represents 18 months of clinical service before testing (17). The specimens were alternated between 5 and 55°C ± 2 according to ISO 11405 (International Standards Organization) recommendations. Dwell time was 15 seconds and the transfer time between the two baths was 5 seconds.

-Marginal adaptation and external interface of base materials/overlying composite evaluations

All specimens were mounted on aluminum stubs, and then coated with gold using a sputter coater (Luxor Benchtop, Nanoscience Instruments, AZ, USA) and examined under a scanning electron microscope (SEM) (JSM-6510LV, JEOL Ltd., Tokyo, Japan) at (×25-500) magnifications to assess the marginal adaptation of each base material at dentin gingival margin. All SEM examination and measurements were performed by one operator having experience with quantitative margin analysis who was blinded to the restorative procedures. The marginal integrity of each base material/gingival dentin was expressed as; percentage of continuous margin (%CM) (whole length of perfect margin (in millimeters)/ (whole length of perfect margin + whole length of imperfect margin) X 100) and maximum gap width (MGW) (µm). The marginal quality was classified according to the criteria” continuous/gap free (or exhibit less than 1 µm gap)” or “discontinuous/gap; exhibit more than 1 µm wide”, according to a well-proven protocol relating margins in gingival dentin consistent with previous studies (18,19).The overall view of margins was examined at ×25 then each part of the base material/gingival dentin interface was further examined at ×200. Finally, parts of detecTable gaps were checked and calculated at ×500. Images were analyzed with image analysis software (SEM Control User Interface Ver 3.10, JEOL Ltd.). All SEM measurements were repeated twice for each specimen. The interface between each base material and overlying resin composite was qualitatively analyzed using SEM at (×200-1500). The criteria for evaluation included presence of gaps, cracks and micro pores at the interface.

-Internal adaptation and internal interface of base materials/overlying composite evaluations

All specimens were fixed vertically to acrylic blocks from their distal side. Each tooth was sectioned longitudinally with a slow speed diamond saw (Isomet 4000-Buehler, Lake Bluff, IL, USA) with water coolant in mesiodistal direction. Each specimen received 2 cuts to produce 3 slices per specimen, and the middle section was 1.0 mm thick. The inner side of the base material/gingival dentin interface of each slice was evaluated for internal adaptation and base materials/overlying composite interfaces using SEM by the same technique and parameters mentioned in marginal adaptation part. The values of the 4 internal surfaces examined were averaged.

-Statistical analysis

The data were statistically analyzed using SPSS (SPSS version 20, IBM, Chicago, IL, USA). The intra-examiner reliability was tested by intraclass correlation coefficient (ICC) calculated for the 2 measurements of each of %CM and MGW data. The %CM values for both external and internal adaptation proved to be normally distributed after they were subjected to the Shapiro–Wilk test, however, MGW values externally and internally were not normally distributed. Two way ANOVA test was used to determine the effect of study variables (base material type and aging condition) and their interaction on %CM followed by Tukey’s post-hoc test (at *p* < 0.05).The Kruskal–Wallis test was used to determine the effect of base material type on both external and internal MGW immediately and after aging, in case of a significant effect, Mann–Whit¬ney U test was used. Finally, Pearson’s and Spearmen correlation coefficients (r) tests were used to correlate both external and internal %CM and MGW values.

## Results

-Results of %CM evaluation

Two-way ANOVA test showed that both study variables significantly affected the %CM values, both externally and internally (*p* < 0.05), the interaction between both variables were also significant (*p* < 0.05). The mean %CM values and standard deviations for all base materials subgroups, externally and internally, are presented in Table 2. For both external and internal %CM values, the immediate subgroups’ values of all materials were higher than aged ones, the difference was significant in RMGI and Bulk Flow groups. Activa and Bulk Flow respectively had the highest values and were not statistically significant from each other. This followed by RMGI and HV-GIC, that were significant than each other in all subgroups except in external aged subgroups. The lowest %CM was for internal aged RMGI subgroup. Pearson correlation between external and internal %CM values showed significant positive relation for all base materials subgroups (r = 0.962, *p* ˂ 0.001).

**Table 2:**
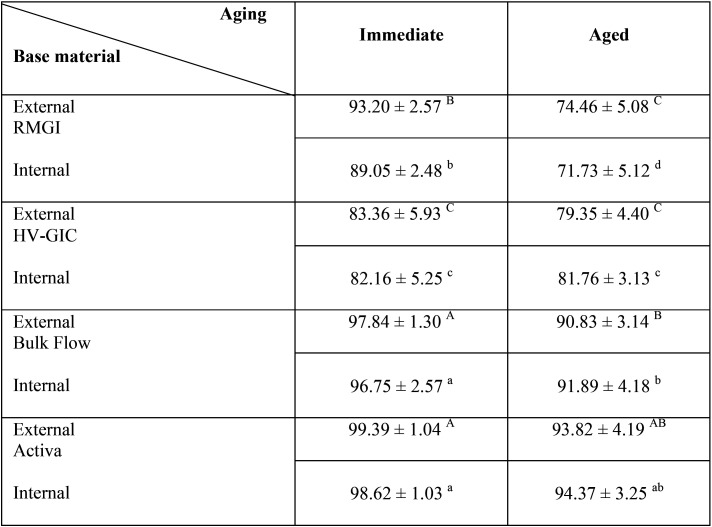
Mean ± SD (%) of % CM values of external and internal marginal adaptation among tested base materials immediately and after aging.

-Results of MGW evaluation 

**Figure 1:**
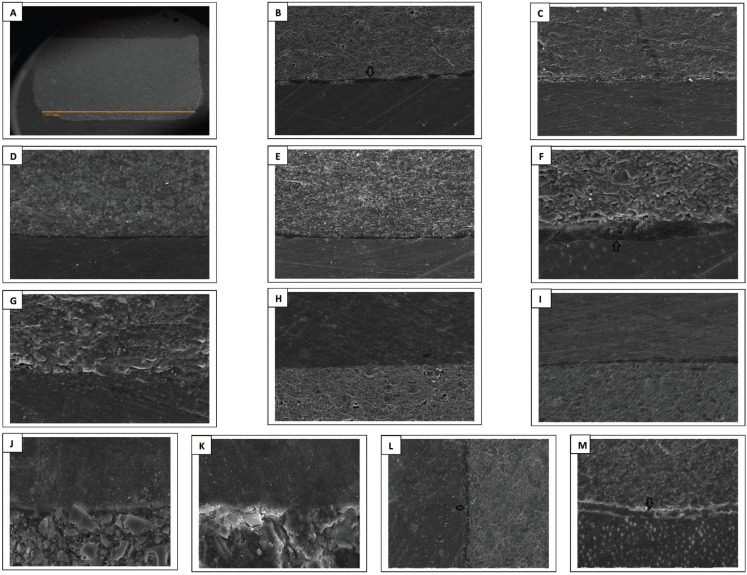
Representative SEM images of external adaptation evaluation of the tested base materials. A: RMGI at ×25 (overall view). B, C D and E: RMGI, HV-GIC, Bulk Flow and Activa//gingival dentin interfaces at ×200 (black arrow in B points to the resin rich layer noticed at RMGI/gingival dentin interface). F and G: RMGI and HV-GIC/gingival dentin interfaces at ×500 (Black arrow in F points to the resin rich layer). H, I, J and K: RMGI and HV-GIC/overlying resin composite interfaces at ×200 for H and I, and at ×1,500 for J and K. L and M: HV-GIC/axial and gingival dentin interfaces at ×200 (Black arrows point to the possible ion exchange zones).

**Figure 2:**
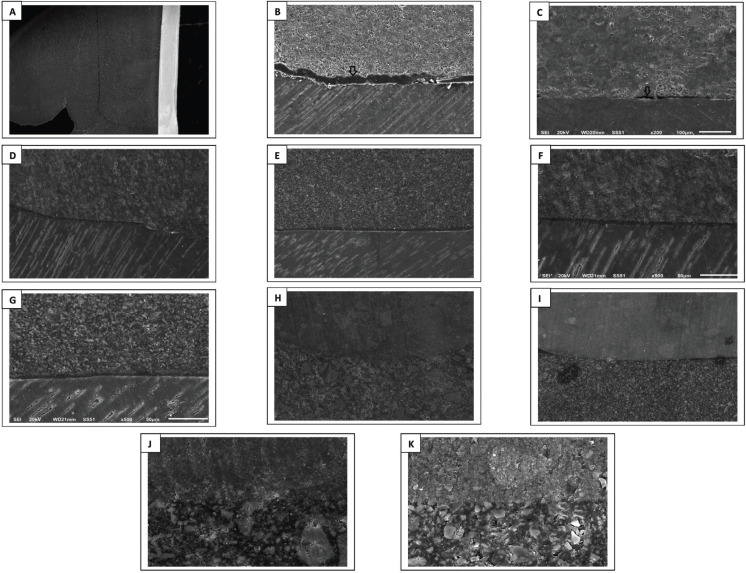
Representative SEM images of internal adaptation evaluation of the tested base materials. A: Bulk Flow at ×25 (overall view). B, C D and E: RMGI, HV-GIC, Bulk Flow and Activa//gingival dentin interfaces at ×200 (black arrows in B point to the resin rich layer and in C point to a marginal gap). F and G: Bulk Flow and Activa/gingival dentin interfaces at ×500. H, I, J and K: Bulk Flow and Activa/overlying resin composite interfaces at ×200 for H and I, and at ×1,500 for J and K.

The results of kruskal Wallis test showed statistical significant differences among each base material’s subgroups MGW values, both externally and internally. Upon performing multiple comparisons of each base material subgroups, externally and internally, all immediate subgroups had less gap widths than aged ones, and the differences were statistical significant in all materials’ groups (*p* < 0.05) (Table 3), except between the external HV-GIC subgroups. Results (as median) of the same subgroups of all base materials’ comparisons for external and internal MGW values separately are presented in Table 4. All base materials showed statistical significance differences between the same subgroups except between all subgroups of Bulk Flow and Activa (*p* > 0.05), which had the least MGW values, both immediately and after aging. Spearman correlation between external and internal MGW values showed significant positive relation between external and internal MGW values for all base materials’ subgroups (r = 0.923, *p* ˂ 0.001). Representative SEM images for external and internal adaptation evaluations and all base materials/overlying composite interfaces with all magnifications are presented in Figs. 1 and 2.

**Table 3:**
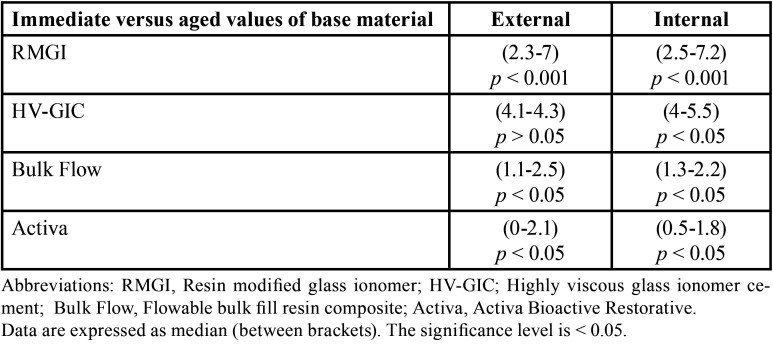
Results of Mann–Whitney U test for comparisons of maximum gap width among immediate and aged subgroups of tested base materials, externally and internally.

**Table 4:**
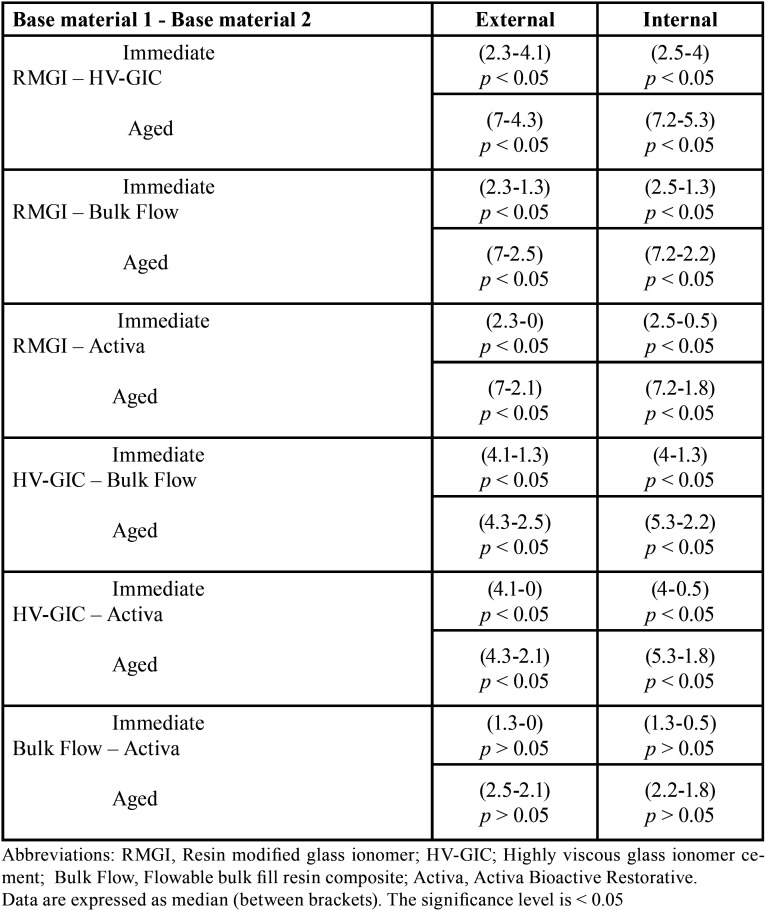
Results of Mann–Whitney U test for comparisons of maximum gap width among tested base materials immediately and after aging, externally and internally.

There were some interesting findings during the evaluation. For the immediate subgroups of both external and internal RMGI/dentin margins, a resin rich layer was noticed with a thickness of 12-30 µm, although no adhesive was applied before RMGI placement (Fig. 1B,F, Fig. 2B). This layer was less evident in the aged subgroups. In addition, during marginal evaluation of HV-GIC aged subgroups, a continuous distinct layer was noticed along the interfacial zone between the HV-GIC group and dentin margins in some of the aged specimens (Fig. 1L,M). It had clearly obvious different morphology than either HV-GIC or dentin.

-Qualitative evaluation for each base material/overlying resin composite interface, externally and internally

The interface between all base materials with overlying resin composite was continuous with no voids, gaps or cracks, externally and internally, even after aging (Figs. 1, 2H-K). Generally, the adhesive thickness, when used, was more in the immediate subgroups than aged ones.

## Discussion

The universal adhesive in this study was used in selective etch mode. Based on previous studies, universal adhesives perform better with dentin when used in self-etch (SE) mode compared to etch and rinse mode (20). There was no resin top coat added on the surface of the GI specimens to simulate the clinical scenario when elevating deep proximal subgingival margin.

Both the type of base material and aging condition affected the marginal integrity results, therefore, the first null hypothesis was rejected. Both Bulk Flow and Activa had the best external and internal adaptation values immediately and after aging. The better marginal integrity of both Bulk Flow and Activa could be related to 2 factors; the materials themselves and the adhesive used. Bulk Flow used is a highly filled flowable material that may lead to a decrease in the volumetric shrinkage and overall polymerization stress (21). This is combined with the low elastic modulus of the material that allowed better flow and ensured better filling for the irregular margins (22). For Activa, the high flow of the material due to the low filler loading, and as experienced during manipulation, can lead to better adherence to the gingival margin (23), in addition, this fluidity could result in improved adaptation to all cavity walls with irregular line and point angles (24).

The current adhesive contains 10-Methacryloyloxydecyl dihydrogen phosphate (10-MDP) monomer that chemically bond to hydroxyapatite crystals forming a nanolayer that further could lead to improved marginal sealing (25). The current adhesive contains Decandiol dimethacrylate (D3MA) and Methacrylated carboxylic acid polymer (MCAP) (26). D3MA enables the reaction of the adhesive with the less polar monomers of resin composite. MCAP is a carboxylic acid functional polymer that bonds to hydroxyapatite (26). The presence of many carboxylic acid groups along a polymeric chain allows multiple bonds to the tooth surface (26).

Both RMGI and HV-GIC had the lowest marginal integrity values. Czarnecka *et al*. (27), reported that the stickiness of RMGI, same used in this study, created difficulties in proper condensation of the material in just small sections, which might cause deteriorated marginal sealing. Furthermore, the high viscosity of the tested HV-GIC can hinder the good penetration and adaptation of the material (28), which may negatively affect its marginal quality. Additionally, the absence of surface protection for HV-GIC may had adversely affected the marginal sealing as was mentioned previously, especially before aging (29).

Aging had a detrimental effect on the marginal integrity of all materials’ groups. Both Bulk Flow and Activa were bonded to gingival dentin using a universal adhesive. During aging of the adhesive interface, a degradation of the hybrid layer usually occurs, especially if simplified adhesives were used due to their hydrophilic properties (16). Such adhesives behave as a semi-permeable membrane and become more susceptible to water absorption that can speed up the hydrolysis and decomposition of interface components (16).

RMGI in the current study had drastic decrease in marginal integrity after long term aging. Vidal *et al*. (30) reported similar results to our findings and explained them by the presence of hydrophilic functional groups in RMGI that can absorb water more easily over time, acting as a plasticizer as may occur by hydrophilic resin monomers in dentin–restoration interfaces, contributing to its degradation and loss of marginal sealing.

The results of the current study showed significant positive correlation between external and internal adaptation values of each base material regardless of the aging condition, thus, the second null hypothesis was accepted. This might indicate possible prediction of the internal adaptation of restorative materials to dentin/cementum margins from the clinical external marginal examinations.

It was reported that the compression applied through the matrix-band on the surface of the resin-based materials can probably cause the filler particles to slide in the organic matrix, and result in accumulation of resin rich layer against the matrix-band (31). Despite the low resin percentage in the used RMGI, the resin rich layer noticed at RMGI/gingival dentin margins in this study could be theorized based on the previous finding; occlusal compression of the RMGI against gingival floor led to resin accumulation against this part of the cavity.

Despite the lack of elemental analysis for the distinct layers at some of the HV-GIC/dentin interfaces, this could be an ion exchange layers. The development of ion-exchange layers usually caused by diffusion of respective cations into the interfacial zone and there react with appropriate anions to form mechanically strong acid resistant structure (32). The formation of these layers in the aged specimens might be one of the causes of the preserved marginal quality of HV-GIC after aging (33). However, further investigations are needed to confirm that.

Based on the current study results, all base materials/overlying resin composite interfaces had a good seal without imperfections or gaps, even after aging, furthermore, there was no difference in the qualitative evaluation of these interfaces, and thus, the third null hypothesis was accepted. RMGI, Bulk Flow and Activa have a resin part in their composition, making the good interface with overlying adhesive and resin composite directly relatively expected. What was interesting is the excellent interface between HV-GIC that has no resin content, with the overlying universal adhesive. Francois *et al*. (14) suggested that there was a chemical bond between a universal adhesive and same HV-GIC used in the current study via interaction between the dihydrogenphosphate group of 10-MDP from the universal adhesive and the calcium ions from the GI matrix. This ionic bonds would result in MDP-calcium salts that are sTable in an aqueous environment.

Although SEM examination has been the backbone of the research conducted on the analysis of resin-dentin interface (18), it is an invasive technique, which considered as a limitation in the present study. Clinical studies concerning non-invasive evaluation of marginal quality for subgingival dentin/cementum margins elevated by different restorative materials are needed.

## Conclusions

Within the limitations of the present study, it may be concluded that: in terms of marginal integrity, both flowable bulk fill resin composite and tested bioactive material might be preferable for proximal dentin gingival margins elevation under direct restoration compared to GI-based materials. All tested base materials/overlying resin composite interface should not be a matter of concern for either researchers or clinicians.
